# Dependence on material choice of degradation of organic solar cells following exposure to humid air

**DOI:** 10.1002/polb.23905

**Published:** 2015-09-16

**Authors:** Tom S. Glen, Nicholas W. Scarratt, Hunan Yi, Ahmed Iraqi, Tao Wang, James Kingsley, Alastair R. Buckley, David G. Lidzey, Athene M. Donald

**Affiliations:** ^1^ Cavendish LaboratoryUniversity of CambridgeJ J Thomson AvenueCambridgeCB3 0HEUnited Kingdom; ^2^ Department of Physics and Astronomy, Hicks BuildingUniversity of SheffieldHounsfield RoadSheffieldS3 7RHUnited Kingdom; ^3^ Department of Chemistry, Dainton BuildingUniversity of SheffieldBrook HillSheffieldS3 7HFUnited Kingdom; ^4^ School of Materials Science and EngineeringWuhan University of TechnologyWuhan430070China; ^5^ Kroto Innovation CentreOssila LimitedBroad LaneSheffieldS3 7HQUnited Kingdom

**Keywords:** degradation, humidity, organic photovoltaics, TEM, water

## Abstract

Electron microscopy has been used to study the degradation of organic solar cells when exposed to humid air. Devices with various different combinations of commonly used organic solar cell hole transport layers and cathode materials have been investigated. In this way the ingress of water and the effect it has on devices could be studied. It was found that calcium and aluminum in the cathode both react with water, causing voids and delamination within the device. The use of poly(3,4‐ethylenedioxythiophene) polystyrene sulfonate (PEDOT:PSS) was found to increase the degradation by easing water ingress into the device. Replacing these materials removed these degradation features. © 2015 The Authors. Journal of Polymer Science Part B: Polymer Physics published by Wiley Periodicals, Inc. J. Polym. Sci., Part B: Polym. Phys. 2016, 54, 216–224

## INTRODUCTION

It is hoped that organic materials will one day provide cheap, flexible, thin film solar cells which can be easily produced using non‐toxic processes and materials.^1^, ^2^ However, such organic photovoltaic (OPV) devices still have low efficiencies compared with other competing technologies and have operational lifetime problems due to sensitivity to ultraviolet light, water, and oxygen.^3^, ^4^ Much research has been focused on increasing efficiencies, but the longevity of these devices is also important and more understanding is needed of the processes involved so that degradation can be minimized.

Degradation of OPV performance can be caused by a number of different factors, due to the multilayered structure and finely balanced morphology of the active layer, each of which can be negatively altered. Encapsulation can be used to improve the lifetime, but adds additional costs to the device fabrication.^5^, ^6^

The effect of air exposure on organic solar devices has been studied previously.^7^, ^8^, ^9^, ^10^, ^11^, ^12^ Increasing evidence suggests that the top electrode is important in regards to degradation.^13^ An oxide layer that forms when aluminum is used as the top cathode has been reported.^7^, ^10^, ^12^ The presence of the polymer poly(3,4‐ethylenedioxythiophene) polystyrene sulfonate (PEDOT:PSS) as the hole transport layer has also been found to enhance degradation in air^10^ compared with devices with alternative hole transport layers^12^ or none at all.^9^

The effect of water ingress has also been studied, and is increasingly thought to be one of the main causes of degradation in organic solar cells.^12^, ^14^, ^15^ It is still not clear whether ingress through grain boundaries^14^, ^16^ or from edge diffusion^12^, ^15^ is more significant. Again, PEDOT:PSS enhances the observed degradation, and this is attributed to its hygroscopity rather than its acidity.^12^

The use of a silver cathode^8^ or a molybdenum oxide (MoO*_x_*) hole transport layer^12^ has been shown to increase device stability with regards to air exposure compared with devices with the more standard aluminum cathode and PEDOT:PSS hole transport layer.

The degradation of ITO/PEDOT:PSS/PCDTBT–PCBM/Ca/Al organic solar cells in humid air has been described in detail previously.^17^ In these devices large bubbles in the PEDOT:PSS were found and smaller but extensive voids formed at the cathode/active layer interface. It was also found that decreasing the grain size of the aluminum increased the rate of degradation. It was proposed that water ingress into the device and reacted with the aluminum and calcium to produce hydrogen. This hydrogen is then thought to cause delamination in the form of bubbles and voids. Smaller grained aluminum was proposed to be more susceptible to void formation due to the different mechanical properties compared with the larger grain film, leading to a poorer adhesion between the layers.

It should be noted, and the motivation for this work, that different combinations of electrodes have been used by other workers^8^, ^12^, ^18^, ^19^, ^20^, ^21^, ^22^ because of improved matching of energy levels and hence presumed efficiency. However, as we will show here, such gains in efficiency may be more than offset by the rapid degradation of the electrodes. Bubbles and delamination seriously compromise improvements in performance.

In this work cross‐sectional transmission electron microscopic (TEM) images, scanning electron microscopic (SEM) images of the top surface and current–voltage measurements of OPV devices during and after the ageing process are taken. Unencapsulated devices with various combinations of cathode and hole transport layer were exposed to humid air in order to accelerate any possible damage caused by water ingress. In this way a greater understanding of water ingress and the effect of water on these systems can be determined. The results presented here reflect the complexity of OPV device degradation and show that layers other than the active layer must also be considered if device stability is to be improved. Indeed, multiple layers are shown to be involved in water‐induced degradation.

## EXPERIMENTAL

### Device Fabrication

Pre‐patterned indium tin oxide (ITO) substrates provided by Ossila Ltd were used for OPV device fabrication. These substrates measure 20 × 15 mm, with six active pixels each of 4 mm^2^. Before fabrication the substrates were cleaned by sonications in various solutions; first with dilute sodium hydroxide (NaOH), then with a Hellmanex^®^ solution, then deionized water, and finally isopropyl alcohol.

The active layer of the devices used in this study was made using a polymer:fullerene blend. The polymer used was poly[*N*−9′‐heptadecanyl‐2,7‐carbazole‐alt‐5,5‐(4′,7′‐di‐2‐thienyl‐2′,1′,3′‐benzothiadiazole)], known as PCDTBT, synthesized by Yi as reported previously.^23^ The electron acceptor used was [6,6]‐phenyl‐C71 butyric acid methyl ester (PC_70_BM), provided by Ossila Ltd. These materials were dissolved in a polymer:PC_70_BM ratio of 1:4 at a concentration of 25 mg/mL in chlorobenzene.

**Figure 1:**
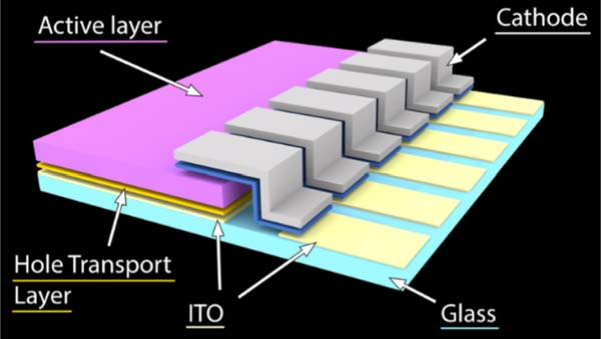
OPV device structure. Photocurrent is collected at the cathode or at the ITO anode. The cathode strip connects the active area to another ITO contact for ease of electrical connection. It consists of an optional calcium interlayer (shown in *blue*) and an aluminum or silver capping layer. The hole transport layer and active layer are wiped away from the ITO connecting with the cathode. The active region of the device is the part with all the layers present, shown in the middle.

The device structure was ITO/hole transport layer/PCDTBT–PC_70_BM/cathode, as shown in the schematic in Figure 1. Two different hole transport layers were used in this study, PEDOT:PSS and molybdenum oxide. PEDOT:PSS was spun cast onto the cleaned ITO substrates in air to form a layer approximately 20 nm thick before being annealed for 5 minutes at 130 °C to remove absorbed moisture. The PEDOT:PSS was then removed from the region of the ITO that made contact with the cathode using a cotton bud dipped in de‐ionized water. Molybdenum oxide was deposited onto the cleaned ITO substrates by thermal evaporation using the evaporation chamber inside a nitrogen filled glovebox.

The PCDTBT–PC_70_BM active layer was spun cast in a glovebox to form a layer approximately 70 nm thick. The active layer and any molybdenum oxide HTL were then removed from the cathode ITO contact using chlorobenzene and a cotton bud.

The cathode was thermally evaporated using the chamber in the glovebox. When calcium was used as an interlayer, an approximately 8 nm thick layer of calcium was deposited by evaporation first before approximately 75 nm of aluminum or silver was evaporated on the top. Finished devices were left unencapsulated and sealed in the glovebox for transportation to be aged or tested.

Most devices in the batch were used to record the change in *J–V* characteristics after devices were aged in a high humidity environment. A few devices were not tested and instead cross‐sectioned and viewed using electron microscopy at various stages of ageing. Each device configuration was fabricated and tested as a separate batch.

### Device Ageing

Devices were aged in high humidity by placing them in a desiccator with a saturated salt solution. Sodium acetate (C_2_H_3_NaO_2_) was used to give a relative humidity of 76%.^24^ Devices were placed in the desiccator for an hour before being removed and tested again. This was repeated for 4 hours, before being left overnight and tested again after a total of 14 hours. Devices aged in low humidity were placed in a desiccator with silica gel, giving a relative humidity of approximately 15%. A third group was left at ambient humidity. During the ageing process devices were left at open circuit, at ambient temperature, and were exposed to the ambient artificial room lighting.

### Electron Microscopy

SEM images of the top surfaces were taken with an incident electron energy of 10 kV using an FEI Philips Dualbeam Quanta 3D FIB. The *in‐situ* lift‐out method, described in detail elsewhere, was used to obtain cross‐sectional TEM samples. These samples were made as thin as possible and all are approximately 70 nm or less, measured approximately using the SEM of the dual‐beam system. Once completed, cross‐sectional specimens were transferred immediately and quickly to an FEI Philips Tecnai 20 and viewed with incident electron energy of 200 kV. ImageJ software was used for any area measurements made from electron micrographs.

### Device Testing

Device *J–V* characteristics were obtained by illuminating the devices under a Newport 9225 1A‐1000 AM1.5 solar simulator, under ambient conditions with the use of a shadow mask to define the active area as 2.12 mm^2^ An NREL calibrated silicon cell was used to calibrate the power output to 100 mW/cm^2^ at 25 °C. A Keithley 237 source meter was used to measure the current as a function of voltage. To eliminate the effect of dead pixels, only the 50% of pixels with the highest performance were used when averaging.

The ageing and testing procedure used in this study does not fit exactly with the suggested ISOS protocols.^25^ It is most similar to the ISOS‐D‐1 shelf test, with the differences being devices were exposed to ambient artificial room lighting while being aged and the humidity was controlled.

## RESULTS AND DISCUSSION

This section is split into two parts. In the first, results obtained from devices with a PEDOT:PSS hole transport layer are discussed. The second part discusses those with molybdenum oxide (MoO*_x_*) as the hole transport layer. Upon exposure to humid air devices with PEDOT:PSS and aluminum/calcium exhibited large bubbles in the PEDOT:PSS and much smaller but extensive voids at the cathode/active layer interface, as was reported previously.^17^ These features were studied for different device configurations.

### PEDOT:PSS as Hole Transport Layer

#### Microscopy

We have studied devices utilizing aluminum, aluminum/calcium, silver and silver/calcium cathodes, and a PEDOT:PSS hole transport layer.

**Figure 2:**
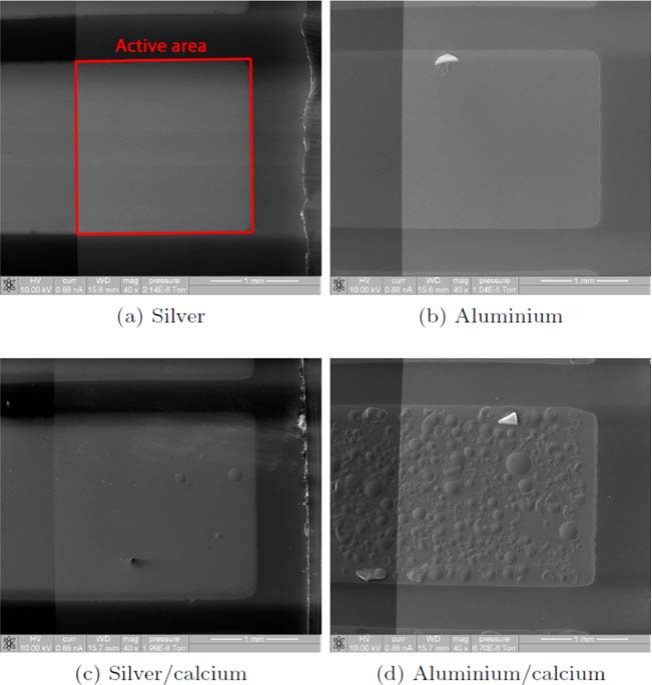
SEM images of devices after 4 hours of exposure to humid air. Bubble defects are much reduced for aluminum without calcium or if silver is used instead of aluminum. No degradation was seen for silver without calcium. The active area is the square region on all four images, as labeled in (a).

Comparison between devices with these cathode structures left in 75% humidity for 4 hours is shown in Figure 2. No defects were seen on devices with silver cathodes [Fig. 2(a)]. Only a few bubble defects were observed on aluminum [Fig. 2(b)] and silver/calcium [Fig. 2(c)] cathode devices. Bubble defects were seen after just 1 hour for aluminum/calcium devices and were seen to spread inward from the edge of the device with time. After 4 hours a large portion of the active area is covered with these defects, as seen in Figure 2(d).

The observed difference in the number of bubbles between devices with aluminum and silver cathodes could be expected as silver is less reactive than aluminum. Silver does not react with water and is stable in air, although it can be corroded by sulfur compounds, which can be found in small quantities in air. The comparison between silver and aluminum cathode devices made in Figure 2(a,b) shows that aluminum must play a role in this degradation mechanism, as the structure of the devices is identical except for the cathode material yet significantly more degradation is observed with the aluminum cathode devices.

The addition of a calcium interlayer causes enhanced degradation for both silver and aluminum cathode devices, therefore showing that the presence of calcium can also contribute to degradation. Both of these findings confirm our speculation that replacing aluminum with silver, removing calcium, or both, will reduce the amount of hydrogen produced in reactions with water, and therefore reduce damage to the device and reduce degradation of performance. The observation of no degradation on the silver cathode device type means that the degradation is not caused by the PEDOT:PSS layer.

**Figure 3:**
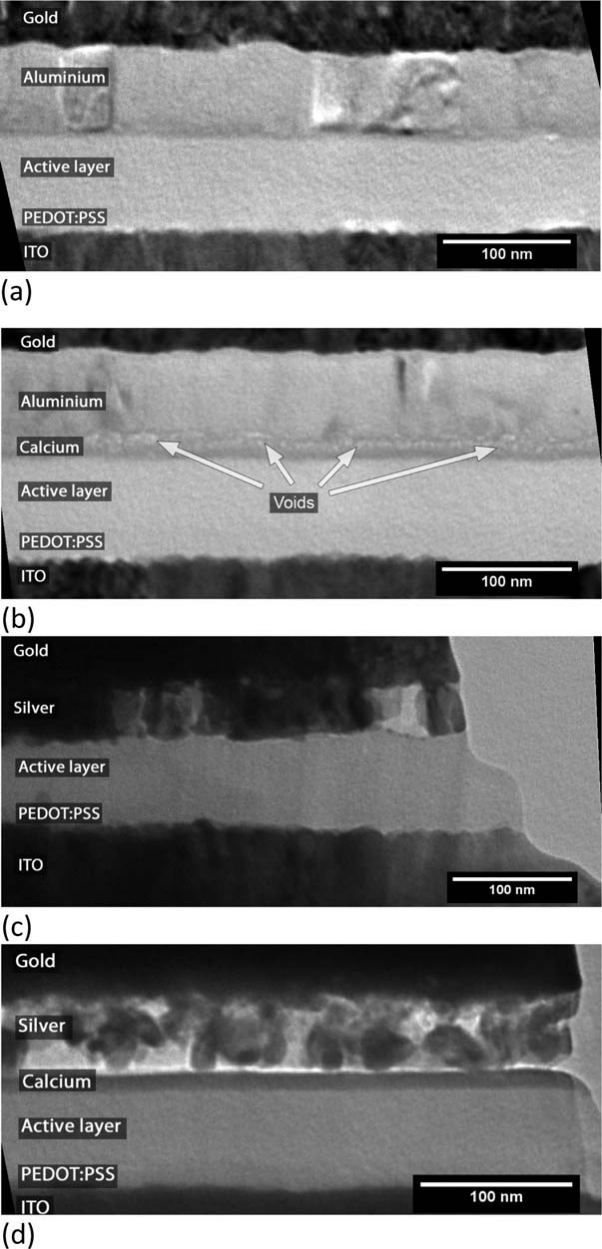
TEM images of void formation at the cathode interface. Far fewer voids are seen with just aluminum (**a**) than when calcium is also used (**b**). No defects are seen at the silver/active layer interface in (**c**), but voids along the silver/calcium interface are seen in (**d**). The *red dashed line* in (d) marks the line intensity profile taken and shown in Figure 4(a).

Figure 3 shows TEM cross‐sectional images taken from silver and aluminum devices with and without calcium after 4 hours of exposure to humid air. These images revealed that the small cathode/active layer interfacial voids were far more prevalent when calcium was used with aluminum than when it was not present, as seen in Figure 3(a,b). The equivalent devices with silver cathodes are shown in Figure 3(c,d). Silver cathodes showed no such voids at the interface when no calcium was present, as shown in Figure 3(c). Some voids are present at the silver/calcium interface [Fig. 3(d)].

**Figure 4:**
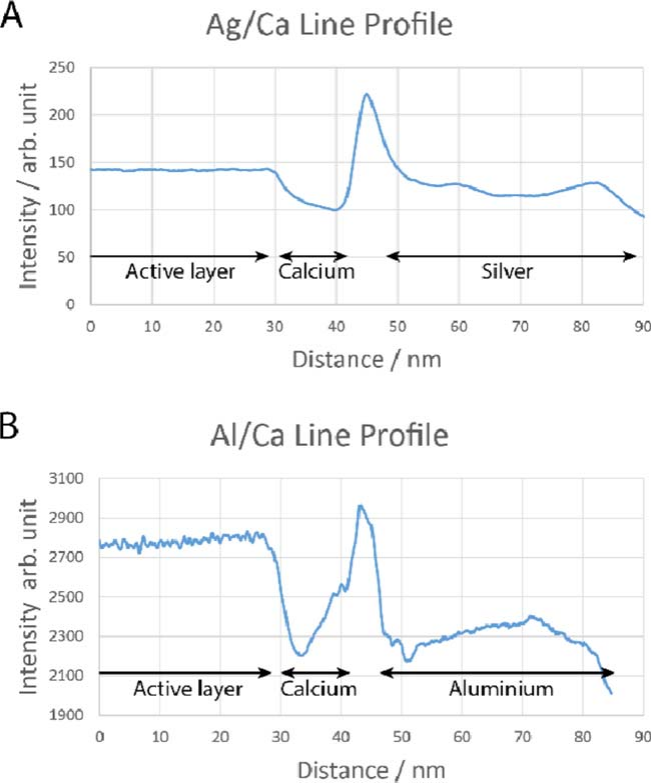
Intensity line profiles across the cathode/active layer interface for silver/calcium (**a**) and aluminum/calcium devices (**b**). The similarity in the intensity peak implies that voids are present at the metal/calcium interface in both.

Producing uniform cross‐sections of devices with silver cathodes using the FIB proved more difficult than those with aluminum. The uneven appearance of the silver layer in Figure 3(d) and to a lesser extent Figure 3(c) was fairly typical, even in fresh devices. Such variations are likely to be due to different crystal orientations between different grains within the layer. It was for this reason that interfacial voids are less obvious on these samples. As such, an intensity line profile was taken, averaged over 300 pixels, across the active layer/cathode interface, as shown by the red dashed line in Figure 3(d). This intensity profile was similar in shape to a profile taken across a void seen at the active layer/cathode interface for an aluminum/calcium device, where the voids are more readily identifiable. In particular the peak between the calcium and metal layers, where the voids are located, is very similar. These profiles are shown in Figure 4. The voids were also seen to increase in size after longer ageing periods, again similar to those seen with aluminum cathodes.

#### Device Testing

Devices were fabricated and their power conversion efficiency (PCE), short circuit current density *J*_sc_, and open circuit voltage (*V*_oc_) was measured after ageing. Different devices were aged in three different environments and tested periodically as they degraded. Some cells were kept in a approximately 76% relative humidity environment, others were kept in the ambient room conditions, while a third group were kept in a dry environment.

**Figure 5:**
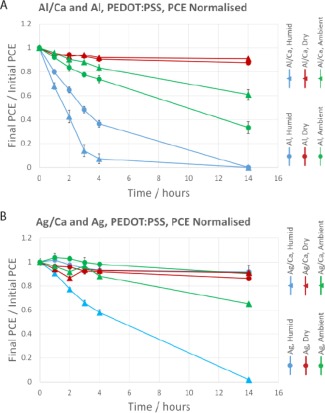
Degradation of normalized PCE over time for devices with PEDOT:PSS HTL. Aluminum/calcium and aluminum cathode devices are shown in (**a**) and silver/calcium and silver cathode devices in (**b**). Average initial PCEs: Al/Ca—4.5%, Al—2.9%, Ag/Ca—4.5%, and Ag—3.5%. Error bars are the standard error of the mean.

Figure 5 shows the change in PCE as the devices were aged. The *J*_sc_ was found to mirror the drops in PCE while the *V*_oc_ remained fairly constant until device failure. The most sensitive devices to humidity were those with aluminum/calcium cathodes. Silver/calcium cathode devices performed better than either aluminum/calcium or aluminum in humidity, but worse than just silver cathodes. The degradation of silver cathode devices was limited and showed no dependence on humidity. Such degradation could be caused by oxygen or changes in the active layer but is beyond the scope of this study.

These *J–V* results can be correlated with the electron microscopy images acquired of aged devices, as devices with the most visible degradation were found to perform worse after exposure to humidity. Silver/calcium and aluminum (no calcium) devices seemed to have similar degradation but it was found that the PCE of silver/calcium devices dropped off more slowly, as can be seen in Figure 5.

### MoO*_x_* as Hole Transport Layer

#### Microscopy

Devices were studied using an aluminum, aluminum/calcium, silver and silver/calcium cathodes, and a MoO*_x_* hole transport layer.

**Figure 6:**
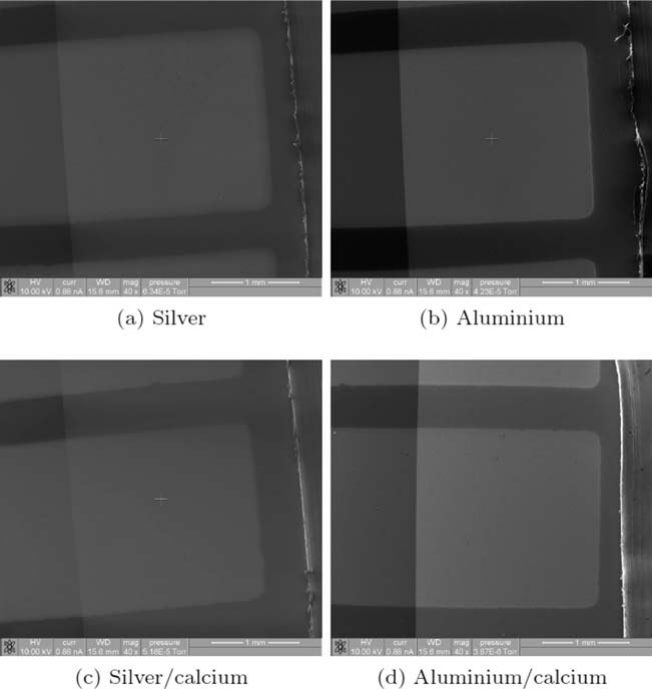
SEM images of MoOx HTL devices after 4 hours of exposure to humid air. No bubble defects were observed for any of the cathode configurations. The active region is the square area.

Comparison between devices with these cathode structures in 75% humidity for 4 hours is shown in Figure 6. No bubble defects were observed after 4 hours for any of the cathode combinations when MoO*_x_* was used. This suggests that the degradation in MoO*_x_* HTL devices is significantly reduced compared with the PEDOT:PSS equivalent shown in Figure 2, where bubble defects are prevalent.

**Figure 7:**
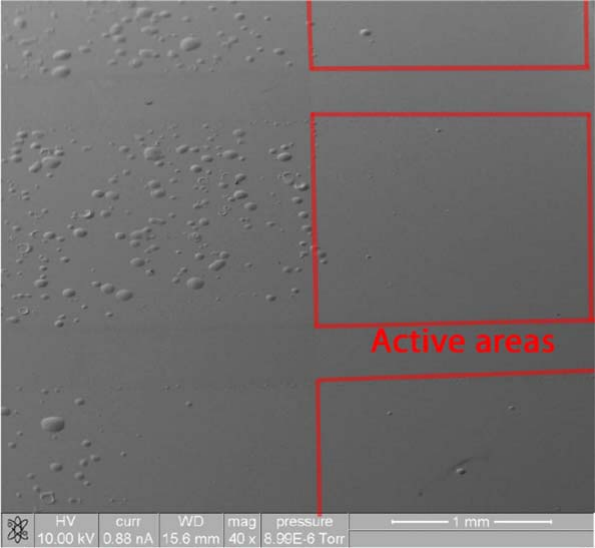
Defects were seen after 14 hours for aluminum/calcium MoO_x_ devices. Pixels are marked in *red*. Region without ITO next to active area is more greatly affected.

After 14 hours of exposure, only devices with aluminum/calcium cathodes had bubble defects, shown in Figure 7. In this image most of the bubble defects are seen in the regions to the left of the active area, although a few are seen in the active areas of the device. It is along this region that the MoO*_x_* and active layer are wiped using a cotton bud dipped in chlorobenzene to make contact with the cathode. Bubble defects are also observed that have formed on the edge of the outer pixels where no MoO*_x_* was deposited due to a misalignment with the evaporation mask. From this it can be deduced that the bubbles are formed most readily in the areas of the device in which no MoO*_x_* is present. This shows that this degradation is dependent on the materials present, a result in agreement with the previous findings. In this case, this dependence could be caused by different material configurations changing the rate of water ingress or the effect of water on the materials in the device.

It is also possible that the different combinations of materials have different adhesive strengths. If one layer is deposited onto another, with the layers having low mutual adhesion, then this interface would be vulnerable to buckling and delamination if any gas or swelling were to be produced in the device. Similar buckling and delamination in carbon or multilayer stacks have been reported elsewhere on other systems after deposition using certain methods or mechanical testing.^26^, ^27^, ^28^ Gas build up would cause a stress build up causing debonding of layers. In other multilayer stacks the formation of these structures is thought to be caused by a well‐defined combination of internal strain, Young's modulus, coating thickness and adhesion energy.^26^ As such, some combinations of materials are likely to be more susceptible to the formation of such structures.

**Figure 8:**
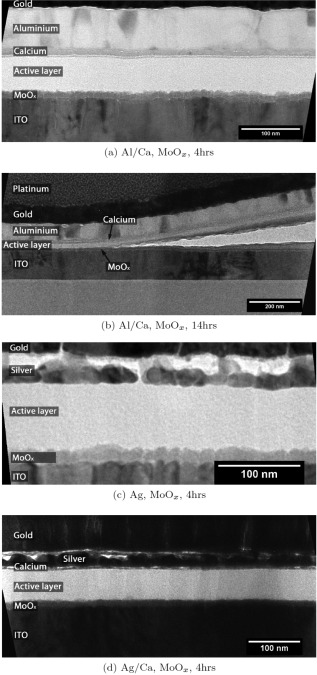
TEM images of devices with MoO*_x_* HTL. (**a**) shows a typical aluminum/calcium interface, with a few small voids noticeable, while (**b**) shows a low magnification image of a bubble defect, showing the delamination occurring at the MoO*_x_*/active layer interface. The silver/active layer interface shown in (**c**) is similar to the PEDOT:PSS equivalent discussed previously, while the void formation at the silver/calcium interface shown in (**d**) seems to be reduced for MoO*_x_* HTL devices.

Cross‐sectional TEM reveals that there are far fewer voids at the cathode/active layer interface after 4 hours (Fig. 8) when MoO*_x_* is used rather than PEDOT:PSS as was shown in Figure 3. This is in agreement with the reduced bubble formation observed using the SEM. Cross‐sectioning a bubble defect reveals that delamination occurs between the MoO*_x_* and active layers, as shown in Figure 8(b).

#### Device Testing

**Figure 9:**
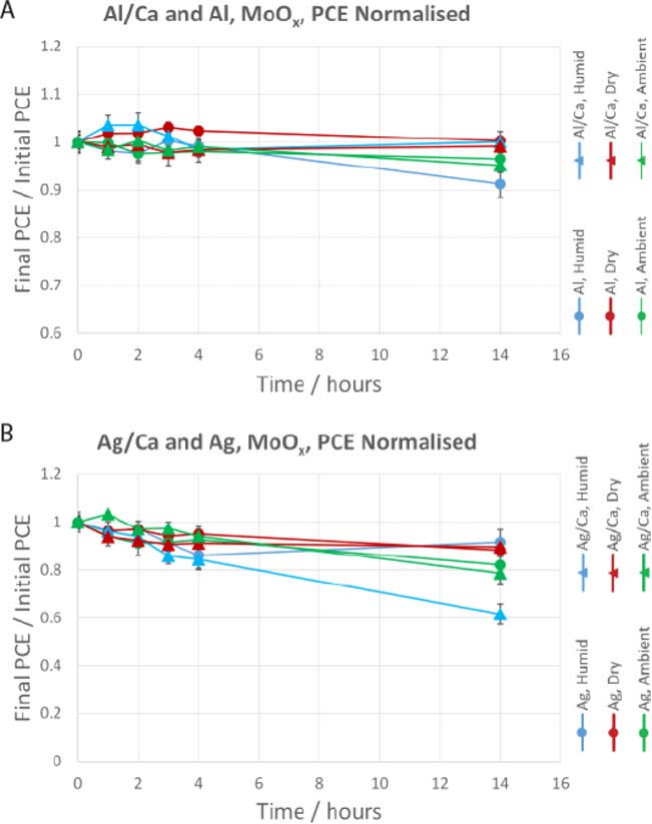
Degradation of normalized PCE over time for devices with MoO*_x_* HTL. Aluminum/calcium and aluminum cathode devices are shown in (**a**) and silver/calcium and silver cathode devices in (**b**). Average initial PCEs: Al/Ca—5.2%, Al—3.7%, Ag/Ca—4.9%, and Ag—3.7%. Error bars are the standard error of the mean.

The change in PCE for the devices with a MoO*_x_* hole transport layer is shown in Figure 9. All devices with MoO*_x_* HTLs were still operational after 14 hours, including those kept in the humid environment. This, again, compares favorably with the PEDOT:PSS devices discussed previously (Fig. 5).

Surprisingly aluminum/calcium devices had a reduced loss in performance compared with devices using an aluminum cathode when MoO*_x_* is used as the HTL. For aluminum and aluminum/calcium cathode devices the *J*_sc_ again mirrored the PCE graph shown in Figure 9(a), with the *V*_oc_ remaining approximately constant. This was the same for silver/calcium, but silver cathodes showed a slight drop in *V*_oc_ when exposed to humidity. Silver cathode devices exhibited slight loss in PCE but it is not clear whether this is caused by humidity. It is clear for silver/calcium devices, however, that these devices performed worse over time than those without calcium when exposed to a humid atmosphere, but degraded at the same rate in dry air.

### Comparison and Discussion

We note that the devices in this study were prepared not encapsulated and were exposed to a high humidity environment to accelerate the ageing process. However, gradual exposure to small amounts of water over a longer period of time may result in different degradation mechanisms. The results reported here are of interest, but a degree of caution is needed when considering degradation of devices under real‐world conditions.

Despite this caveat, by comparing the presence of bubbles and voids with *J–V* characteristics over time in humid air for devices with varied combinations of hole transport layers and cathodes, it is hoped that some insight into water ingress and the effects of water in devices can be gained.

PEDOT:PSS devices were found to be more susceptible to moisture than those with MoO*_x_* HTLs. PCEs reduced more rapidly and a larger number of bubbles and voids were observed. All MoO*_x_* devices were still operating at 60% or more of their initial PCE after a 14 hours exposure to humid air. This compares favorably with PEDOT:PSS devices, some of which were only at approximately 15% of their original PCE after just 3 hours of humid air exposure.

Aluminum cathode devices were also more susceptible to moisture than those with a silver cathode. The exception to this was aluminum/calcium cathode devices with MoO*_x_* HTL that performed better than the silver/calcium equivalent. Silver (without calcium) cathode devices were apparently unaffected by moisture. Their PCE after 14 hours of exposure to humid air was approximately 90% of the original PCE; a value no different to the same devices left in ambient and dry conditions. No bubbles or voids were seen in these devices. The choice of MoO*_x_* or PEDOT:PSS as the HTL did not change this.

In general the addition of calcium leads to faster degradation regardless of HTL or top cathode material, although aluminum/calcium with MoO*_x_* HTL again proves an exception.

It would seem from this evidence that the cause of the decrease in efficiency in the devices exposed to humid air is a reaction with water. This can occur with either calcium or aluminum and is facilitated by the presence of PEDOT:PSS as the HTL. PEDOT:PSS is known to be hygroscopic.^29^ We propose that water ingresses through the PEDOT:PSS layer, diffusing laterally into the active region of the device where it can permeate into and through the other layers. At the cathode interface, water will react with any calcium or aluminum present to produce hydrogen, which then causes delamination and voids to be formed within the device. Removal of calcium or the use of silver reduces the formation of these features as silver does not react with water. The use of silver without calcium as a cathode prevented the formation of these features completely.

Substituting MoO*_x_* for PEDOT:PSS reduces this process. While still hygroscopic,^30^, ^31^ MoO*_x_* is thought to be less hygroscopic than PEDOT:PSS,^9^, ^12^ so water ingress into the device is reduced. Once the amount of water ingressing into devices is reduced, we believe that other degradation mechanisms or factors may become more significant. The unexpected result seen with devices with aluminum and MoO*_x_*, which degraded faster than equivalent devices with silver cathodes, could arise from the grain size difference between aluminum and silver films that may be more significant when the lateral water ingress is reduced due to the less hygroscopic HTL. Smaller grained aluminum has been found to be more susceptible to void formation, possibly due to the different mechanical properties compared with the larger grain film, leading to poorer adhesion; or perhaps due to water ingress through the cathode being easier through the smaller grains, and perhaps a similar difference is seen here between the larger grained aluminum and the smaller grained silver devices, although further investigation is required to identify the exact mechanism. Despite this, it can be deduced from this study that PEDOT:PSS is not the cause of this water induced degradation as some degradation is still seen in devices with no PEDOT:PSS, but it has been shown to facilitate it and enhance the degradation.

As water ingresses from the edges of the device, the lateral design is important as previously demonstrated.^15^ Larger area pixels would mean water would take longer to affect the entire pixel, while wiping away any unnecessary hygroscopic HTLs exposed to air should also reduce water intake into the device.

Other research groups have been able to fabricate devices with long operational lifetimes using PEDOT:PSS as the HTL by using additives in the PEDOT:PSS or thorough device encapsulation.^32^, ^33^ The TEM images and results presented in this article highlight why such mitigation is necessary.

## CONCLUSIONS

This work has shown that the material choice made in the design of organic solar cells has a large impact on the degradation of devices when exposed to humid air. We have proposed that water ingresses through the hole transport layer, permeates through the layers of the device and reacts with any calcium or aluminum present in the cathode. Both the hole transport layer and cathode materials have been shown to have an effect on this degradation. Different material choice, lateral design or thorough encapsulation methods can all play a role in reducing the effect of water on organic solar cells. If different materials are to be used, hygroscopicity and adhesion strength of any subsequent layers are important properties to consider if lifetime is to be maximized.
